# Extrapontine Myelinolysis of Osmotic Demyelination Syndrome in a Case of Postoperative Suprasellar Arachnoid Cyst

**DOI:** 10.1155/2012/679257

**Published:** 2012-12-27

**Authors:** Peng Zhao, Xuyi Zong, Xinsheng Wang, Yazhuo Zhang

**Affiliations:** ^1^Department of Neurosurgery, Beijing Tiantan Hospital, Capital Medical University, Beijing 100050, China; ^2^Beijing Neurosurgical Institute, Capital Medical University, No. 6 Tiantan Xili, Chongwen District, Beijing 100050, China

## Abstract

The extrapontine myelinolysis of osmotic demyelination syndrome (ODS) is a well-known but uncommon disorder of the central nervous system. Although the mechanism is not fully understood and the treatment is controversial, hyponatremia is probably considered to be the main pathophysiological basis. There are few reports of ODS caused by a sellar lesion. Here we present a case of suprasellar arachnoid cyst that developed extrapontine myelinolysis of ODS after a neuroendoscopic treatment procedure. It is suggested that patients with suprasellar lesions are at risk of developing extrapontine myelinolysis of ODS and correction of hyponatremia in these cases should be closely monitored.

## 1. Introduction

Osmotic demyelination syndrome (ODS) is characterized by partial destruction of myelin sheath at the basis pontis (central pontine myelinolysis, CPM) or outside the pons (extrapontine myelinolysis, EPM) [1]. Clinical features of the syndrome show atypical neurological deficits according to the affected area. And the main causes include alcoholic, malnourished, chronically debilitated status [2]. For the lesions in the sellar area such as pituitary adenoma, germ cell tumor, and craniopharyngioma, there are also distributed reports [3]. Although the mechanism of ODS is not fully understood, the hyponatremia is considered to be the main pathophysiological process [4]. We report a case of suprasellar arachnoid cyst presenting with extrapontine myelinolysis of ODS after neuroendoscopic procedure. To our knowledge, there is no report of a similar case in the literature. Therefore it is attributed to the clinical practice.

## 2. Case Report

**Figure 1:**
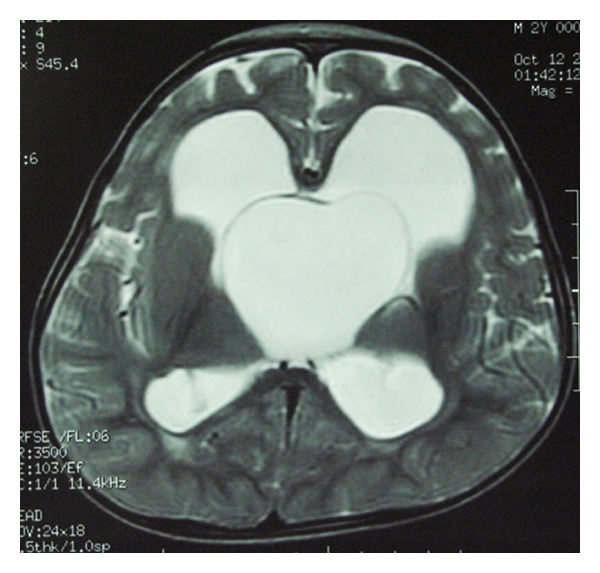
The preoperative of MRI image showed a cyst lesion at the suprasellar cistern which compressed the third ventricle (T2 weighted image).

A 3-year-old boy was admitted to hospital with inability to walk for 18 months. Past history showed that he underwent ventricle-peritoneal shunt operation 1.5 years old for hydrocephalus and he had no renal and liver malfunction. The visual, hearing, and verbal function was normal. Physical examination showed weakness of both lower limbs. The pituitary-adrenal axis test was normal before operation. Magnetic resonance imaging (MRI) of the brain revealed a cyst lesion at the suprasellar cistern which compressed the third ventricle (Figure 1). After a complete of medical evaluation, he received a neuroendoscopic operation to remove the suprasellar lesion. During the procedure, the cyst wall was partially excised and at the same time endoscopic third ventriculostomy was performed for the reconstruction of CSF circulation.

**Figure 2:**
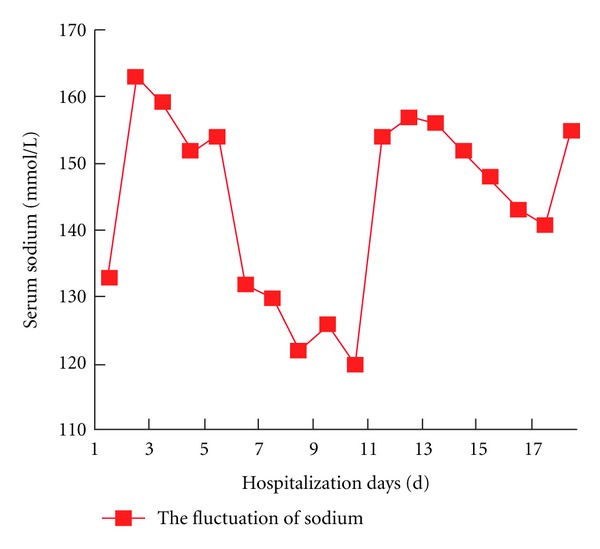
The line graph showed the serum sodium concentration changing with the postoperative time.

**Figure 3:**
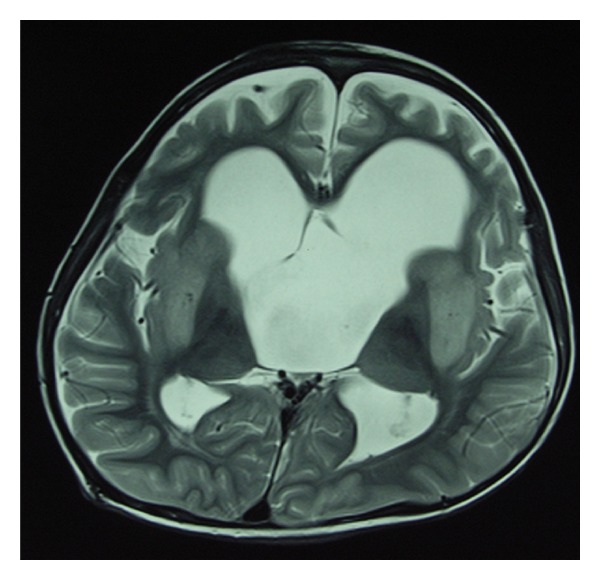
The postoperative MRI image of the patient showed total resection of the cyst lesion, but there existed symmetrical lesions at basal ganglia area (T2 weighted image).

After operation, the patient recovered well. But blood biochemical examination showed that the sodium level was 160 mmol/L (135–145 mmol/L) immediately after operation. Intravenous fluid to limit the sodium level was given. One day later, the blood exam showed that the level went down to the normal range. On day 5, the boy suffered accidental persistent epilepsy status although the prophylaxis antiepileptic drugs were used. After given conservative treatment, his general epileptic status was controlled. The serum sodium concentration was 120 mmol/L at that time. Serum sodium was corrected then and it still fluctuated between 120 and 126 mmol/L. On day 11, the serum sodium concentration went to 155 mmol/L suddenly. All the sodium changing level was drawn on Figure 2. The patient suffered the generalized dystonia and deteriorated dysarthria as well as dysphagia afterwards. Neurological examination showed generalized brisk deep tendon reflexes. On that day, postoperative MRI examination was performed. The imaging showed symmetrical hypersignal intensity at the caudate and putamen with pallidal sparing in T2WI compatible with extrapontine myelinolysis, comparing with the preoperative MRI which showed no such changes (Figure 3). He suffered extrapontine myelinolysis of ODS confirmed by clinical features and image findings. Afterwards the patient underwent a series symptomatic treatment and the serum sodium concentration was corrected to the normal level on postoperative day 17. His condition improved but abnormal movement persisted. The pathological result showed arachnoid cyst. After 3-month rehabilitation exercise, generalized dystonia was markedly improved and he resumed his activity of daily living. 

## 3. Discussion

We describe a case of suprasellar arachnoid cyst developing extrapontine myelinolysis of ODS after operation. It is well known that ODS is associated with hyponatremia closely [4, 5]. Although Adams and his colleagues first described the central pontine myelinolysis (CPM) concept in 1959, the mechanism of ODS is not clear until now [1]. But many reports suggested that the ODS was probably caused by demyelination of the nerve sheath from the rapid change of serum osmotic pressure [6]. The correlation between ODS and rapid correction hyponatremia was documented in clinical as well as animal experimental studies [5]. Kengne et al. reported that abrupt osmotic changes during the rapid correction of chronic hyponatremia resulted in demyelinative brain lesions [7], but the sequence of events linking rapid osmotic changes to myelin loss was not yet understood. He designed a rat model of osmotic demyelination syndrome which showed the death of astrocyte after rapid correction of hyponatremia, delineating the regions of future myelin loss. Perhaps astrocyte death caused a disruption of the astrocyte-oligodendrocyte network, which predicted clinical manifestations and the outcome of osmotic demyelination. In this case, the chronic hyponatremia occurred after operation although the correction was given regularly. And quick change of serum sodium from low to high level could induce ODS. Therefore why the patient suffered the chronic hyponatremia should follow the trail of the neuroendoscopic operation.

Suprasellar arachnoid cysts derive from the sellar area and compress the third ventricle causing hydrocephalus. Srimanee et al. described two cases of sellar lesions with generalized dystonia and MRI results were compatible with EPM [8]. So he recommended careful corticosteroid replacement therapy for the sellar region and correction of hyponatremia as well as a close monitoring of serum sodium were the important issue for the prevention of ODS. In our case, the arachnoid cyst located around the sellar, hypothalamus, and pituitary gland. The water-sodium balance metabolism was easily affected. According to the postoperative treatment, the reason for the extrapontine myelinolysis perhaps was the infusing imbalance of the electrolyte of the serum sodium which led to hyponatremia. Tsutsumi et al. reported a 3-year-old girl presented with ODS after craniopharyngioma operation for the serum sodium concentration fluctuating rapidly. The patient suffered disturbance of consciousness with spastic paraparesis. The depressed mental status persisted and she remained in a vegetative state [3]. From the author's experience, one must care about the surrounding structures such as the hypothalamus, pituitary gland, and other sellar areas to avoid additional damage during a neuroendoscopic procedure. Meanwhile, postoperative serum sodium monitoring must be cared about for pituitary adenoma, craniopharyngioma, suprasellar arachnoid cyst, Rathke's cyst, and so forth. 

Overcorrection hyponatremia is the main cause for CPM and/or EPM if the correcting velocity surpasses 12 mmol/L/24 h or 20 mmol/L/48 h [3]. Most authors agree that the correction of hyponatraemia is not in excess of 8 mmol/L/day for chronic hyponatremia [4]. Treatment of ODS is mainly supportive and symptomatic [9]. MRI could not predict the final outcome as well and some cases had persistent MRI abnormality in spite of complete clinical recovery. The overall prognosis of ODS varies from complete recovery, minimal, to severe disability and death. The prognostic outcomes of movement disorders related to EPM were also varied from resolution of the syndrome within three months to permanent abnormal movements. Kallakatta and his colleagues reported the outcome and factors predicting prognosis in osmotic demyelination syndrome in 25 patients [9]. Eleven (46%) cases had a favorable outcome at a mean follow-up of 2.2 ± 2.5 years. But 13 cases had a poor outcome. So he suggested that higher GCS scores, better scores in functional scales in hospital, less severe hyponatraemia, and absence of superadded hypokalemia could predict favorable outcome. Tsutsumi and his colleagues reported about 50% of patients with ODS died within 2 weeks and 90% within 6 months, and survivors remained little sequelae [3]. In our case, the boy suffered only abnormal movement which persisted with the follow-up.

According to our knowledge, the present case is the first for the suprasellar arachnoid cyst to develop extrapontine myelinolysis of osmotic demyelination syndrome. Care must be taken during sellar region operations to reduce the damage to the nearby anatomy, and postoperative correction of hyponatremia in these cases should be closely monitored.
